# Another two new aphid species of the genus *Cryptomyzus* Oestlund, 1922 (Hemiptera, Aphididae, Macrosiphini) from Kazakhstan

**DOI:** 10.3897/zookeys.1270.158304

**Published:** 2026-02-25

**Authors:** Rustem Kh. Kadyrbekov, Aizhan M. Tleppaeva, Sergey V. Kolov

**Affiliations:** 1 West Kazakhstan University, N. Nazarbaev avenue 162, Uralsk, 0900000, Kazakhstan Institute of Zoology, Ministry of Sciences and Higher Education of Kazakhstan Republic Almaty Kazakhstan; 2 Institute of Zoology, Ministry of Sciences and Higher Education of Kazakhstan Republic, Academgorodok, Al-Farabi avenue, 93, Almaty, 050060, Kazakhstan West Kazakhstan University Uralsk Kazakhstan

**Keywords:** Fauna, genus, keys, new species, Palearctic, systematic

## Abstract

Two new species of the genus *Cryptomyzus* living on Lamiaceae are described from specimens preserved in the collection of the Institute of Zoology of the Republic of Kazakhstan (Almaty). Cryptomyzus (Cryptomyzus) saryarkensis**sp. nov**. living on the ground parts of *Phlomoides tuberosa* in Saryarka region of the Kazakh Uplands in North Kazakhstan and C. (C.) nepetaphilus**sp. nov**. living on the ground parts of *Nepeta cataria* in Zhongar Alatau mountain system in south-eastern Kazakhstan. All measurements were taken on a Bel Photonics BIO 3T light microscope. *Cryptomyzus* (*C.*) saryarkensis**sp. nov**. is closest to C. (C.) maudamanti Guldemond, 1990 and C. (C.) galeopsidis (Kaltenbach, 1843). *Cryptomyzus* (*C.*) nepetaphilus**sp. nov**. is related to C. (C.) ribis (Linnaeus, 1758). Identification keys to related species of *Cryptomyzus* are provided. Two centres of species diversity of the genus *Cryptomyzus* are clearly visible in Europe (6 species) and Central Asia (9). *Cryptomyzus* (*C.*) ribis (Linnaeus, 1758), C. (C.) korschelti Bӧrner, 1938, C. (C.) alboapicalis (Theobald, 1916), and C. (C.) galeopsidis (Kaltenbach, 1843) are distributed much more widely. *Cryptomyzus* (*C.*) taoi Hille Ris Lambers, 1963 is known from Pakistan, India, Russian Far East, and Japan, and C. (C.) behboudii Remaudière & Davatchi, 1961 is known from Turkey and Iran.

## Introduction

*Cryptomyzus* is a Palearctic genus, currently with 19 species in the world fauna (Favret 2024). It is divided into four subgenera: *Ampullosiphon* Heikinheimo, 1955 (1 species), *Alataumyzus* Kadyrbekov, 1993 (1 species), *Cryptomyzus* Oestlund, 1922 (15 species), and *Phlomimyzus* Narzikulov & Daniyarova, 1979 (2 species). Species of this genus with known life cycle either alternate between species of *Ribes* (Grossulaceae) and species of herbaceous plants from Lamiaceae or are monoecious on species of Lamiaceae (Blackman and Eastop 2006, 2020).

Examination of materials collected in 2002 in Saryarka region of the Kazakh Uplands in North Kazakhstan and in 2017 in Zhongar Alatau mountain system in the south-east revealed two *Cryptomyzus* species new to science, which are described here. Keys for distinguishing apterous viviparous females of new taxa from related species and a key to the apterous viviparous females of the genus *Cryptomyzus* Oestlund, 1922 of the world (updated from the key by Kadyrbekov (2021)) are provided.

## Material and methods

Original microscope slides were prepared by A.M. Tleppaeva using coniferous balsam as mounting fluid (Kadyrbekov 2014). The specimens were examined using a Bel Photonics BIO 3T light microscope. Aphid identifications were done with reference to authoritatively identified material from the collection of the Institute of Zoology of Ministry of Sciences and Higher Education of Kazakhstan (IZRK), Almaty. Holotypes and paratypes of newly described species are deposited in the collection of the IZRK (Almaty, Kazakhstan). Micromed 3 U3 light microscope with Basler Powerpack 12.4 MP digital camera was used for the photomicrographs, which have been taken and mounted by S.V. Kolov.

Identification keys to apterous viviparous females of the world *Cryptomyzus* fauna were compiled (Kadyrbekov 2021). Descriptions of some *Cryptomyzus* species were also studied (Hille Ris Lambers 1953; Guldemond 1990; Kadyrbekov 1993).

All measurements are given in millimetres. Plant taxonomical names were verified according to POWO (2024). Following abbreviations are used: **BL** – body length with cauda, **ANT** – total antennae length, **ANT III** – length of III antennal segment, **ANT IV** – length of IV antennal segment, **ANT V** – length of V antennal segment, **ANT VIb** – length of base of VI antennal segment, **ANT IIIbd** – basal diameter of III antennal segment, **PT** – length of processus terminalis, **URS** – length of ultimate rostral segment, **SIPH** – siphunculus length, **CAUDA** – cauda length, **HT II** – length of II segment of hind tarsus, **Longest setae on ANT III** – setae length, **Longest setae on ABD TERG III** – setae length, setae length, **ABD TERG VIII** – setae length, **ANT/BL** – length of antennae to body length with cauda, **PT/ANT VI b** – length of processus terminalis to length of base of sixth antennal segment, **ANT III/ANT VI** – length of III antennal segment to length of VI antennal segment, **Longest setae on ANT III/–ANT IIIbd** – longest setae on ANT III to basal diameter of III antennal segment, **Cephalic frontal setae/–ANT IIIbd** – cephalic frontal setae to basal diameter of III antennal segment, **Longest setae on ABD TERG III/–ANT IIIbd** – longest setae on ABD TERG III to basal diameter of III antennal segment, **Longest setae on ABD TERG VIII/–ANT IIIbd** – longest setae on ABD TERG VIII to basal diameter of III antennal segment, **URS/HT II** – length of ultimate rostral segment to length of II segment of hind tarsus, **SIPH/BL** – siphunculus length to body length with cauda, **SIPH/CAUDA** – siphunculus length to cauda length.

## Results

### Cryptomyzus saryarkensis sp. nov.

Taxon classificationAnimaliaHemipteraAphididae

FEDBD3D4-B7A9-5695-AEB3-405191AE77B8

https://zoobank.org/FEA01124-0A9D-4773-A5C3-C6E1289B89B9

#### Description.

**Figure 1.**
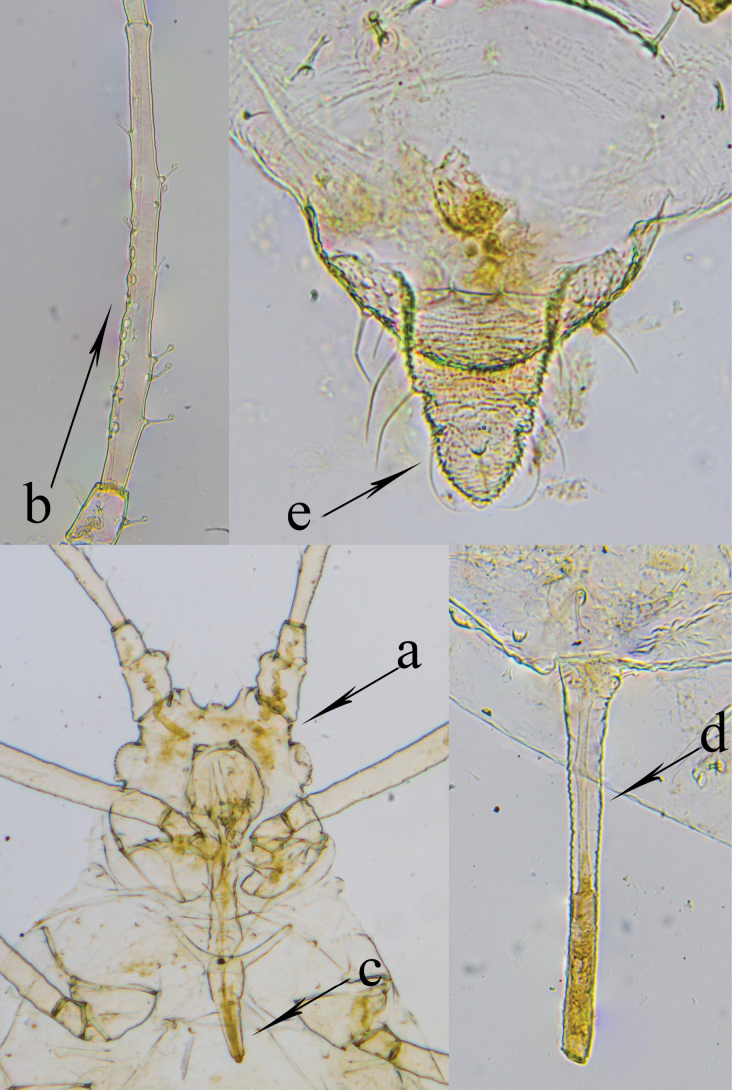
*Cryptomyzus saryarkensis* sp. nov. **a**. Head; **b**. Antennal segment III; **c**. Ultimate rostral segment; **d**. Siphunculus; **e**. Cauda.

**Apterous viviparous female**, from 11 specimens (Fig. 1, Table 1). In life: body white with green markings, eyes reddish. On slide: body and appendages pale without dark parts apart from pale brown tarsi. Body elliptical. Cuticle wrinkled in places. Frontal groove moderately deep, 0.15–0.22 between apices of antennal tubercles (Fig. 1a). Antennal tubercles distinct and divergent. Median frontal tubercle well developed, quadrate. Cephalic hairs long, thickened, capitate or funnel-shaped. Antennae six-segmented. First antennal segment with a large protuberance on inner side at apex and bears 4–5 setae. Numbers of setae on II and III antennal segments are II – 5, III – 4–7. Antennal segment III with 5–13 secondary rhinaria (Fig. 1b). Setae on antennal segment III longer than its diameter at base, slightly capitate. Rostrum reaches beyond bases of hind coxae; ultimate rostral segment long, slender (Fig. 1c), with 2–4 accessory setae. Siphunculi straight, with slightly widened bases and small indistinct flanges (Fig. 1d). Cauda bluntly triangular or helmet-shaped, with 5 long, pointed setae (Fig. 1e). Dorsal setae on abdominal tergites II–V capitate or funnel-shaped. Numbers of setae on abdominal tergites: III – 7–8, VIII – 4–6. Marginal tubercles absent. Genital plate broadly oval, with 2–4 discal setae and 6–10 posterior setae. Legs long. Femur with capitate setae, hind tibia with pointed setae at apex. First tarsal segments with 3:3:2 setae.

**Table 1.** Measurements of *Cryptomyzus nepetaphilus* sp. nov. and *C. saryarkensis* sp. nov.

Measurements	*C. saryarkensis* sp. nov.	*C. nepetaphilus* sp. nov.
	Apterous viviparous females (*n* = 11)	Apterous viviparous females (*n* = 4)
BL	1.95–2.36	2.06–2.21
ANT	2.89–3.54	2.56–2.63
ANT III	0.58–0.68	0.52–0.56
ANT IV	0.48–0.63	0.36–0.42
ANT V	0.43–0.53	0.31–0.35
ANT VIb	0.10–0.13	0.10–0.11
ANT IIIbd	0.029–0.035	0.027–0.029
PT	0.97–1.39	0.99–1.06
Longest setae on ANT III	0.035–0.040	0.023–0.029
Longest frontal setae	0.046–0.052	0.081–0.104
URS	0.14–0.15	0.15–0.16
HT II	0.08–0.10	0.09–0.10
Longest setae on ABD TERG III	0.056–0.058	0.115–0.127
Longest setae on ABD TERG VIII	0.046–0.052	0.115–0.127
SIPH	0.31–0.39	0.39–0.43
CAUDA	0.10–0.15	0.12–0.14
Ratios:		
ANT/BL	1.39–1.59	1.12–1.16
PT/ANT VIb	10.50–12.00	9.50–10.21
ANT III/ANT VI	0.42–0.59	0.45–0.52
Longest setae on ANT III/ANT III BD	1.00–1.27	0.80–1.00
Cephalic frontal setae/–ANT III BD	1.31–1.59	2.79–3.59
Longest setae on ABD TERG III/–ANT IIIbd	1.66–2.00	3.97–4.38
Longest setae on ABD TERG VIII/–ANT IIIbd	1.31–1.59	2.79–3.59
SIPH/BL	0.15–0.18	0.18–0.20
SIPH/CAUDA	2.22–3.75	2.85–3.50

#### Types.

***Holotype***: • apterous viviparous female, slide no 3005, Kazakhstan, Akmola region, “Burabai” natural park, Zhukey lake environs, Berkutty mounting, *Phlomoides tuberosa*, 23 July 2002, R. Kadyrbekov leg. (Institute of Zoology, Almaty, Kazakhstan). ***Paratypes***: • 10 apterous; same place and date.

#### Etymology.

The new species is named after Saryarka region of the Kazakh Uplands.

#### Biology.

This aphid lives on the undersides of leaves of *Phlomoides tuberosa* (L.) Moench (Lamiaceae). It is not attended by ants. The life cycle is unknown.

#### Systematic relationships.

New species belongs to the group of species with setae of antennal segment III exceeding its diameter at base and having fewer than 15 setae on abdominal tergites I–IV (Kadyrbekov 2021). It is closest to C. (C.) maudamanti Guldemond and C. (C.) galeopsidis in having the proportion of siphunculi to body length 0.15 or more. It differs from both species in having straight lines, expanded only at the bases of the siphunculi, PT/ANT VIb = 10.50–12.00 (vs 0.90–10.00) and URS/HT II = 1.41–1.70 (vs 0.90–1.20).

### Cryptomyzus nepetaphilus sp. nov.

Taxon classificationAnimaliaHemipteraAphididae

A0403A54-DB7C-5293-9357-9C72FF7A7A0B

https://zoobank.org/1C75AEC5-2E64-4C2E-BD87-A741A83936CB

#### Description.

**Figure 2.**
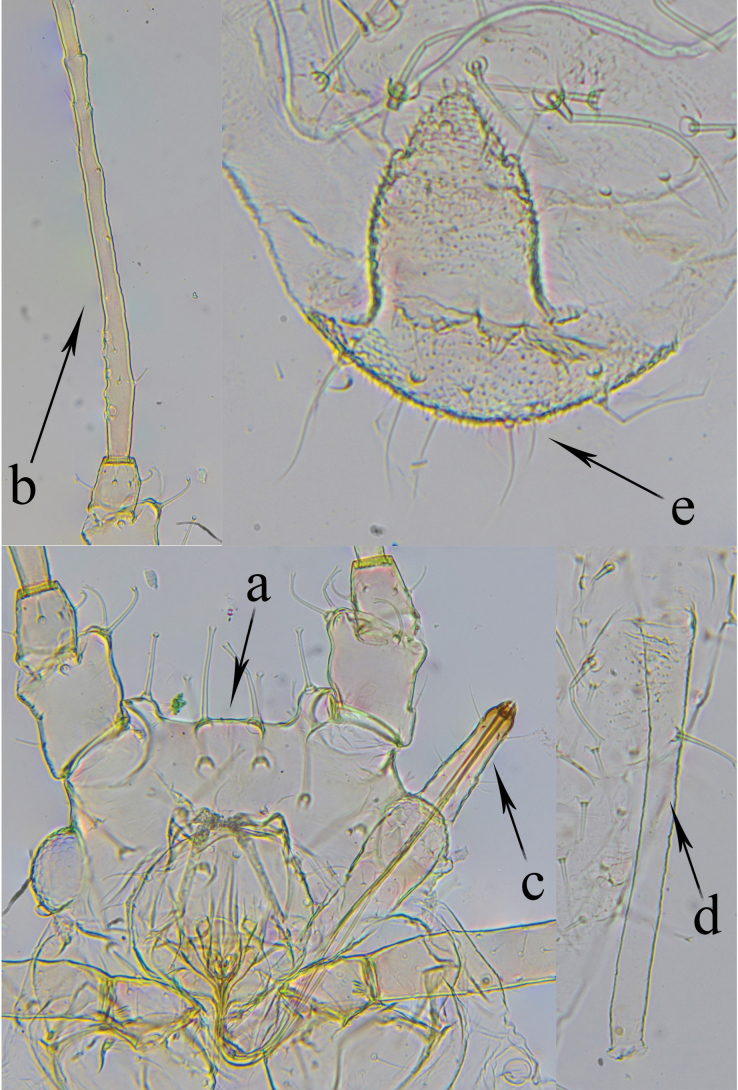
*Cryptomyzus nepetaphilus* sp. nov. **a**. Head; **b**. Antennal segment III; **c**. Ultimate rostral sFegment; **d**. Siphunculus; **e**. Cauda.

**Apterous viviparous femal**e, from 4 specimens (Fig. 2, Table 1). In life: body white with green markings, eyes reddish. On slide: body and appendages pale without dark parts apart from pale brown tarsi. Body elliptical. Cuticle thick, wrinkled in places. Frontal groove moderately deep, 0.16–0.17 distance between apices of antennal tubercles (Fig. 2a). Antennal tubercles distinct and divergent. Median frontal tubercle well developed, quadrate. Cephalic setae long, thickened, capitate or funnel-shaped. Antennae six-segmented. First antennal segment with a large protuberance on inner side at apex and bears 4–5 setae. Numbers of setae on antennal segments II and III are II – 4, III – 3–4. Basal part of antennal segment III with 5–7 secondary rhinaria (Fig. 2b). Setae on antennal segment III shorter than or equal to its diameter at base, slightly capitate. Rostrum reaches beyond bases of hind coxae; ultimate rostral segment long, slender (Fig. 2c), and bears 6–8 accessory setae. Siphunculi subcylindrical, with small, indistinct flanges (Fig. 2d). Cauda bluntly triangular or helmet-shaped, with 5–6 long, pointed setae (Fig. 2e). Dorsal setae on II–V abdominal tergites are capitate or funnel-shaped. Numbers of setae on abdominal tergites: III – 10–12, VIII – 6–8. Marginal tubercles are absent. Genital plate is broadly oval, with 2–4 discal setae and 7–9 posterior setae. Legs are long. Femur with capitate setae, hind tibia with pointed setae at apex. First tarsal segments with 3:3:2 setae.

#### Types.

***Holotype***: • apterous viviparous female, slide no 5752, Kazakhstan, Zhetysu region, Zhongar Alatau mounting system, “Zhongar-Alatau” natural park, 8 km to east from Topolevka small town, “Osinovaja” kordon, H–1250 m, *Nepeta cataria*, 26 July 2017, R. Kadyrbekov leg. (Institute of Zoology, Almaty, Kazakhstan). ***Paratypes***: • 3 apterous, same place and date.

#### Etymology.

The new species is named after it host plant.

#### Biology.

This aphid lives on the undersides of leaves of *Nepeta cataria* L. (Lamiaceae). It is not attended by ants. The life cycle is unknown.

#### Systematic relationships.

This new species belongs to the group of species with setae on antennal segment III, which are shorter than or equal to its diameter at the base and with straight, unswollen siphunculi (Kadyrbekov 2021). It differs from *C. alatavicus* in having ANT III/ANT VI T = 0.42–0.59 (vs 0.68–0.76) and PT/ANT VIb = 9.50–10.21 (vs 6.30–7.30). *Cryptomyzus nepetaphilus* sp. nov. differs from C. (C.) ribis in the proportion of the Longest setae on ABD TERG III/–ANT IIIbd (not more 3 and 3.97–4.38) and SIPH/BL = 0.18–0.20 (vs 0.20–0.25).

##### Key to the apterous viviparous females of the genus *Cryptomyzus* Oestlund, 1922 of the world (updated from the key by Kadyrbekov 2021)

**Table:** 

1	Setae ABD TERG I–IV and median frontal and antennal tubercles shorter ANT IIIbd, inconspicuously capitate. Alternates between *Ribes rubrum* L. and *Galeopsis*, *Stachys*, *Lamium*	**C. (Ampullosiphon) stachydis (Heikinheimo, 1955)**
–	Setae ABD TERG I–IV and median frontal and antennal tubercles usually much longer ANT IIIbd, either on thick bases and capitate or needle-shaped	**2**
2	Setae ABD TERG I–IV and median frontal and antennal tubercles long and needle-shaped. SIPH without distinct flanges	**3**
–	Setae ABD TERG I–IV, median frontal and antennal tubercles capitate. SIPH with distinct flanges	**4**
3	Median frontal tubercle absent. PT/ANT VIb 7.0–7.5. III ANT with about 3 secondary rhinaria. URS/HT II 1.5. ABD TERG III–V with 12–16 setae. On *Phlomis canescens* Regel	**C. (Phlomimyzus) tadzhikistanicus Narzikulov & Daniyarova, 1979**
–	Median frontal tubercle developed. PT/ANT VIb 7.5–9.5. III ANT with 7–19 secondary rhinaria. URS/HT II 1.0–1.1. ABD TERG III–V with 18–27 setae. On *Leonurus turkestanicus* V.I. Krecz. & Kuprian. and occasionally *Lamium album* L.	**C. (P.) multipilosus Kadyrbekov, 2000**
4	Secondary rhinaria occur on ANT III–IV and sometimes on ANT V. Dark medial pleural, and marginal sclerites developed on all ABD TERG. SIPH dark brown, cylindrical, with large flanges. Cauda trapezoid. Alternates between *Ribes saxatile* Pall. and *Eriophyton lamiiflorum* (Rupr.) Brӓuchler	**C. (Alataumyzus) malkovskii Kadyrbekov, 1993**
–	Secondary rhinaria occur only on ANT III–IV. Dark medial pleural, and marginal sclerites absent from all ABD TERG. SIPH pale, cylindrical or swollen, with small flanges. Cauda bluntly triangular or helmet-shaped	**5**
5	Longest setae ANT III usually shorter ANT IIIbd and shorter than the ANT I setae	**6**
–	Longest setae on ANT III longer ANT IIIbd, about the same length as those the ANT I setae	**15**
6	URS/HT II not less 1.8	**7**
–	URS/HT II not more 1.7	**9**
7	ANT III with 0–2 secondary rhinaria. ANT IV shorter than ANT V. Longest setae on ANT III/ANT III BD 0.5–0.8. On *Phlomis*	**8**
–	ANT III with 6–8 secondary rhinaria. ANT IV longer than ANT V. Longest setae on ANT III/ANT III BD. Longest setae on ANT III/ANT III BD 0.9–1.0. Alternates between *Ribes alpinum* L. and *Clinopodium vulgare* L., *Betonica officinalis* L.	**C. (Cryptomyzus) heinzei Hille Ris Lambers, 1953**
8	URS/HT II 2.7–3.0 and URS/–ANT VIb 2.8–3.3. SIPH/CAUDA 2.9–3.5 and SIPH/BL 0.17–0.20. H II/ANT VIb 1.0–1.1. On *Phlomis olivieri* Benth	**C. (C.) behboudii Remaudière & Davatchi, 1961**
–	URS/HT II 1.8–2.0 and URS/–ANT VIb 1.4–1.6. SIPH/CAUDA 3.7–5.0 and SIPH/BL 0.23–0.30. H II/ANT VIb 0.75–0.85. On *Phlomis salicifolia* Regel	**C. (C.) sairamugamicus Kadyrbekov, 2021**
9	SIPH approximately cylindrical	**10**
–	SIPH distinctly swollen	**12**
10	ANT III/ANT VI 0.46–0.53. ANT III with 5–7 secondary rhinaria. PT/ANT VIb 8.8–10.5. SIPH/CAUDA 2.5–3.5 and SIPH/BL 0.18–0.24	**11**
–	ANT III/ANT VI 0.68–0.76. ANT III with 10–37 secondary rhinaria. PT/ANT VIb 6.3–7.3. SIPH/CAUDA 2.0–2.3 and SIPH/BL 0.16–0.18. On *Scutellaria*	**C. (C.) alatavicus Kadyrbekov, 1993**
11	Longest setae on ABD TERG III/–ANT IIIbd not more 3. SIPH/BL 0.20–0.25 in norm more 0.20. Alternates between *Ribes* and *Stachys*	**C. (C.) ribis (Linnaeus, 1758)**
–	Longest setae on ABD TERG III/–ANT IIIbd 3.97–4.38. SIPH/BL 0.18–0.20. On *Nepeta cataria* L.	**C. (C.) nepetaphilus sp. nov**.
12	SIPH/CAUDA not more 4.5. URS with not less than 9 accessory setae	**13**
–	SIPH/CAUDA not less 5. URS with not more than 9 accessory setae	**14**
13	ANT III/ANT VI 0.45–0.55. PT/ANT VIb 8.0–10.5. ABD TERG III–V with 12–18 setae. Alternates between *Ribes alpinum* L., *R. heterotrichum* C.A. Mey., *R.* sp. and *Stachys*	**C. (C.) korschelti Bӧrner, 1938**
–	ANT III/ANT VI 0.59–0.67. PT/ANT VIb 6.0–8.5. ABD TERG III–V with 6–8 setae. On *Ballota nigra* L. and occasionally *Lamium album* L.	**C. (C.) ballotae Hille Ris Lambers, 1953**
14	ANT III/ANT VI 0.43–0.54. PT/ANT VIb 5.8–8.2. URS with 4–6 accessory setae. SIPH/CAUDA 5.1–6.5 and SIPH/BL 0.30–0.35. On *Betonica betonicifolia* (Rupr. Ex O. Fedtsch. & B. Fedtsch.) Sennikov	**C. (C.) karzhantavicus Kadyrbekov, 2021**
–	ANT III/ANT VI 0.57–0.59. PT/ANT VIb 9.0–9.1. URS with 7–9 accessory setae. SIPH/CAUDA 5.0–5.1 and SIPH/BL 0.20–0.25. On *Marrubium supinum* L., *Lamium*, *Leonurus sibiricus* L., *Phlomoides bracteosa* (Royle ex Benth) Kamelin & Makhm.	**C. (C.) taoi Hille Ris Lambers, 1963**
15	ABD TERG I–IV each with 12–20 setae	**16**
–	ABD TERG I–IV each with 5–14 setae	**18**
16	ANT III with 1–4 secondary rhinaria. URS with 6 accessory setae. On *Eriophyton*	**C. (C.) transiliensis Kadyrbekov, 1993**
–	ANT III with more than 5 secondary rhinaria. URS with not more than 4 accessory hairs	**17**
17	ANT III with 11–22 secondary rhinaria. URS/HT II 1.4–1.5. SIPH/CAUDA 1.5–2.1. PT/ANT VIb less than 9t. On *Leonurus cardiaca* L.	**C. (C.) leonuri Bozhko, 1961**
–	ANT III with 5–15 secondary rhinaria. URS/HT IIURS/HT II 1.2. SIPH/CAUDA 1.0–1.5. PT/ANT VIb 10.1–11.0. On *Lamium album* L.	**C. (C.) alboapicalis (Theobald, 1916)**
18	SIPH/BL 0.12–0.15. On *Lamium maculatum* L.	**C. (C.) ulmeri (Börner, 1952)**
–	SIPH/BL more than 0.15	**19**
19	SIPH subcylindrical. PT/ANT VIb 10.50–12.00. URS/HT II 1.41–1.70. On *Phlomoides tuberosa*	**C. (C.) saryarkensis sp. nov**.
–	SIPH in norm swollen. PT/ANT VIb not more 10.00. URS/HT II not more 1.20. On other plants	**20**
20	Rostrum extending backward to hind coxae. ABD TERG I–IV each, with 8–14 setae. SIPH always swollen. Alternates between *Ribes rubrum* L. and *Galeobdolon luteum* L.	**C. (C.) maudamanti Guldemond, 1990**
–	Rostrum shorter. ABD TERG I–IV each, with 4–11 setae. SIPH sometimes not swollen. Alternates between *Ribes nigrum* L., *R. rubrum* L. and *Galeopsis*	**C. (C.) galeopsidis (Kaltenbach, 1843) complex**

## Discussion

There are now 21 species of aphids in the genus *Cryptomyzus* worldwide, including the two new species. Because two new species were described, the existing identification key to species by Kadyrbekov (2021) was modified.

Two centres of species diversity of the genus *Cryptomyzus* are clearly visible in Europe (6 species) and Central Asia (9). *Cryptomyzus* (*C.*) ribis, C. (C.) korschelti, C. (C.) alboapicalis, C. (C.) galeopsidis are distributed much more widely. *Cryptomyzus* (*C.*) taoi is known from Pakistan, India, and the Far East, and C. (C.) behboudii is known from Turkey and Iran.

## Supplementary Material

XML Treatment for Cryptomyzus saryarkensis

XML Treatment for Cryptomyzus nepetaphilus

## Additional information

### Use of AI

No use of AI was reported.

### Data availability

All of the data that support the findings of this study are available in the main text.
